# In this month’s Bulletin

**DOI:** 10.2471/BLT.21.001221

**Published:** 2021-12-01

In the editorial section, Helen Clark et al. (846) introduce a handbook on social participation for universal health coverage. Wilson M Were and Anshu Banerjee (847) describe the revisions of WHO recommendations on managing hypoglycaemia in children. Angela K Shen et al. (848) explain how to ensure optimal supplies of vaccines for COVID-19. 

Samba Sow talks to Andréia Azevedo Soares (853–854) about the need for regional approaches to medical product development, including treatments for mild and moderate COVID-19. Soares (851–852) also reports on WHO’s role in meeting unprecedented demand for effective supply chains in emergency response situations.

## Algeria, Nigeria, Rwanda, Senegal and South Africa 

### Increased local vaccine production

Anna Mia Ekström et al. (910–912) introduce recent efforts to expand capacity.

## Brazil, India, Kenya

### Developing global guidance on human milk banking

Mirriam Tyebally Fang et al. (892–900) explore an unregulated industry.

## Kenya 

### The price tag for maternal survival

Kenneth Juma et al. (855–864) estimate catastrophic expenditure and costs of treatment. 

## Kenya, Malawi, South Sudan, Zimbabwe

### Treatment-adjusted prevalence 

Beth A Tippett Barr et al. (874–882) propose a new indicator for HIV testing programmes.

## Nigeria

### Improving hospital care 

Justina O Seyi-Olajide et al. (883–891) pilot the national surgical, obstetric, anaesthesia and nursing plan.

## Western Pacific Region 

### Implementing cross-sectoral nutrition policies 

Erica Reeve et al. (865–873) analyse policy-makers’ perspectives.

## Global

### Post COVID-19 condition

Janet V Diaz et al. (901–903) list current research questions. 

### Treating hypoglycaemia in children

Helena Hildenwall and Fatsani Ngwalangwa (904–906) call for updated guidance.

### Medical products of human origin

Paul Ashford et al. (907–909) detail issues of ethics, traceability and biovigilance.

